#  “WE WANT TO PAY AND WE WANT TO STAY”: OLDER ADULTS MANAGING PROPERTY TAX BURDEN IN A GROWING URBAN COUNTY

**DOI:** 10.1093/geroni/igac059.1046

**Published:** 2022-12-20

**Authors:** Anthony Traver, Katie White, Marisa Sheldon, Holly Dabelko-Schoeny, Bethany Sanders

**Affiliations:** The Ohio State University, Columbus, Ohio, United States; The Ohio State University, Columbus, Ohio, United States; Ohio State University, Columbus, Ohio, United States; Ohio State University, Columbus, Ohio, United States; Franklin County Auditor's Office, Columbus, Ohio, United States

## Abstract

Aging in place is a goal for many older adults. As many older adults own their homes, strategies designed to promote aging in place must account for threats to the financial sustainability of ownership and occupancy later in life. One such threat is property taxes, which have risen substantially in many metropolitan areas over the last decade as home values soar. Property tax relief programs offered by state and local governments are designed to ease the housing cost burden of older adults. Yet, recent research indicates that such programs do little to ensure affordability for low-income homeowners. This study reviewed local property tax relief programs and interviewed local older adult homeowners and housing professionals to understand the circumstances of older adult homeowners in one growing U.S. County. Four major themes emerged from the interviews: housing market dynamics, personal finances, local housing resources, and wellbeing. Results indicate that unaffordability is a growing concern among older adult homeowners and services providers alike. Current property tax relief programs are thought to do little to reduce the cost burden posed by property taxes. Implications for social policy include expanding eligibility criteria and indexing the benefit to a local economic metric so that the relief remains relevant in areas with dynamic markets. Implications for practitioners include understanding the property tax relief programs in one’s area and referring clients when appropriate.

